# Prevalence, risk factors and seasonal variations of different Enteropathogens in Lebanese hospitalized children with acute gastroenteritis

**DOI:** 10.1186/s12887-019-1513-8

**Published:** 2019-04-30

**Authors:** Ali Salami, Hadi Fakih, Mohamed Chakkour, Lamis Salloum, Hisham F. Bahmad, Ghassan Ghssein

**Affiliations:** 1 Rammal Hassan Rammal Research Laboratory, Physio-toxicity (PhyTox) Research Group, Lebanese University, Faculty of Sciences (V), Nabatieh, Lebanon; 2 Department of Pediatrics, Faculty of Medical Sciences, Lebanese University, Beirut, Lebanon; 3 Department of Biology, Faculty of Arts and Sciences, American University of Beirut, Beirut, Lebanon; 4 Faculty of Medicine, Beirut Arab University, Beirut, Lebanon; 5 Department of Anatomy, Cell Biology, and Physiological Sciences, Faculty of Medicine, American University of Beirut, Beirut, Lebanon

**Keywords:** Acute gastroenteritis, *E. histolytica*, Unidentified enteropathogens, Pediatric, Lebanon

## Abstract

**Background:**

Acute gastroenteritis (AGE) is a major cause of pediatric morbidity and mortality around the world. It remains a frequent reason for infection-related admissions to emergency units among all age groups. Following the Syrian refugee crisis and insufficient clean water in our region, we sought to assess the etiological and epidemiological factors pertaining to AGE in South Lebanon.

**Methods:**

In this multi-center cross sectional clinical study, we analyzed the demographic, clinical and laboratory data of 619 Lebanese children from the age of 1 month to 5 years old who were admitted with AGE to pediatrics departments of three tertiary care centers in South Lebanon.

**Results:**

Our results revealed that males had a higher incidence of AGE (57.3%) than females. Enteropathogens were identified in 332/619 (53.6%) patients. Single pathogens were found in 294/619 (47.5%) patients, distributed as follows: *Entamoeba histolytica* in 172/619 (27.8%) patients, rotavirus in 84/619 (13.6%), and adenovirus in 38/619 (6.1%). Mixed co-pathogens were identified in 38/619 (6.1%) patients. Analyzing the clinical manifestations indicated that *E. histolytica* caused the most severe AGE. In addition, children who received rotavirus vaccine were significantly less prone to rotavirus infection.

**Conclusions:**

Our findings alluded to the high prevalence of *E. histolytica* and other unidentified enteropathogens as major potential causes of pediatric AGE in hospitalized Lebanese children. This should drive us to widen our diagnostic panel by adopting new diagnostic techniques other than the routinely used ones (particularly specific for the pathogenic amoeba *E. histolytica* and for the unidentified enteropathogens), and to improve health services in this unfortunate area of the world where insanitary water supplies and lack of personal hygiene represent a major problem.

## Background

Acute Gastroenteritis (AGE) is a common pediatric illness. In the Middle East region and Lebanon specifically, AGE persists as the second major cause of pediatric mortality and morbidity following acute lower respiratory tract infections [1, 2] and remains a frequent form of presentation due to infectious causes to the emergency units among all age groups [3–5]. Consequently, diarrheal disease is one of the major causes of death globally, where it mostly affects youngsters in undeveloped countries and represents a significant cause of morbidity among children under the age of five in developing countries [6, 7].

In addition to diarrhea, other major symptoms of AGE have been reported to consequently lead to increase in morbidity in severe cases, including vomiting, nausea, weight loss, abdominal pain and dehydration [3, 7]. Gastroenteritis annually affects 3 to 5 billion children worldwide and is responsible for 12% of deaths in children less than 5 years old every year [8]. In developed countries, 1 in 25 children below 5 years of age is diagnosed with AGE [4]; for instance, more than 5 million cases of pediatric AGE are diagnosed in Canada every year [9].

There are several etiologies for AGE, including bacterial, viral, and parasitic enteropathogens. Globally, rotavirus is considered the major cause of infantile AGE [3]. It is considered one of the most significant causes of diarrhea during the first years of life [10]. It was estimated that 440,000 children deaths occur worldwide every year due to rotavirus infections before the release of the rotavirus vaccine [11]. In 2011, data from the coordinated global network for rotavirus surveillance of the World Health Organization (WHO) showed that 37–53% of children hospitalized with diarrhea were infected with rotavirus in regions where vaccination has not been broadly applied [12]. In Lebanon, particularly, previous studies showed a prevalence of 27.7 and 30.6% of rotavirus [13, 14]. Nowadays, rotavirus vaccination is widely available for children in almost all countries and it is highly recommended by physicians [15].

Other than rotavirus, *Entamoeba histolytica* - an intestinal protozoan parasite - is associated with diarrheal diseases, especially human amoebiasis, with a global health concern mainly in developing countries. It is a leading cause of death from parasites around the world and is responsible for more than 50 million infected cases every year, among which 40,000–110,000 patients eventually die [16, 17]. In fact, *E. histolytica* was listed as the second highest priority parasite by the National Institute of Health and Infectious Diseases in the United States [18]. A previous study in Beirut, Lebanon, showed that 22.3% of the cases hospitalized with AGE were infected with *E. histolytica* [14].

Nevertheless, regardless of being a global morbidity and mortality issue among children, AGE preventive measures are achievable via implementing personal and food hygiene, usage of sanitized water, applying vaccination against potential AGE causing viruses and bacteria, and advocating breastfeeding and appropriate nutrition. Such measures and others can heavily prevent the spread of the disease [19].

A recent study performed by our team in South Lebanon evaluated common causes of AGE among hospitalized children during the period of summer 2014. Results from this study indicated that 40.4% of all hospitalized cases in children were due to AGE, with rotavirus and *E. histolytica* being the major identified disease-causing pathogens [20]. To widen our knowledge regarding the incidence, age distribution, etiologies, AGE incidence throughout the different months of the year for each pathogen involved, protective factors, and correlation between AGE causes and severity of the disease among hospitalized children in Southern Lebanon, we performed this current study with a larger sample size, to cover most of the South district. In this multi-center study, 3058 Lebanese children admitted to the pediatrics departments were enrolled, among which 619 were diagnosed with AGE. Frequency and etiology of infectious gastroenteritis was then determined using the available routine laboratory tests.

## Methods

### Patients’ selection

During a one-year period, from the 1st of January 2017 until the 31st of December 2017, we collected and analyzed clinical, demographic and laboratory data of 619 Lebanese hospitalized children, aged between 1 month and 5 years old (60 months old), with acute gastroenteritis (AGE) who were admitted to pediatrics departments of three tertiary care centers (2 governmental and 1 private) in South Lebanon.

Patients included in this study were hospitalized children with AGE or diarrhea, defined as the occurrence of three or more of loose or liquid stools per day (or more frequent passage than is normal for the individual) [19]. We excluded from this study: children with chronic diarrhea, immunodeficiency, malnutrition and those with multiple malformations, since these parameters can negatively affect the length of hospitalization and the severity of the disease which may constitute a disruption in the analysis of our results.

### Clinical variables

Data were collected as follow:

i) Demographic data including: age, gender, date of diagnosis, breast feeding, type of drinking water, housewife mother, family size and the vaccination history to determine if any dose of the two rotavirus vaccines (Rotarix from GlaxoSmithKline Biologicals, Rixensart, Belgium; or Rotateq from Merck Sharp & Dohme Corp, Whitestation, NJ, USA) that are available and approved in Lebanon, was given.
ii) Clinical data including: AGE signs such as fever, diarrhea, vomiting, dehydration, and blood and mucus in stool, in addition to the duration of hospitalization and the calculation of the index of severity “Vesikari Score” [21].
iii) Laboratory findings including: blood levels of WBCs, RBCs, hemoglobin (HGB), hematocrit (HCT), blood sugar (BS), and C-reactive protein (CRP), in addition to the results of stool analysis such as microscopy for ova and parasites, searching for *E. histolytica* by the trichrome stain technique. Although WHO states that *E. histolytica* stool antigen detection test is more specific for the pathogenic amoeba *E. histolytica* than the classic stool ova and parasite examination, the latter was the one still utilized in almost all healthcare centers in South Lebanon, including the three tertiary healthcare centers in our study, which reflects the importance of improving health services in this unfortunate area of the world (South Lebanon). Laboratory findings also included the quick identification tests “rapid tests” for adenovirus (CerTest; Biotec, Zaragoza, Spain) and rotavirus (CerTest), and if available the results of bacterial coproculture.

### Laboratory methods and studies

Fresh stool samples were acquired and analyzed, once received by the laboratory and within less than one hour, for the presence of infectious agents as previously described in a study from our group [20]. In brief, rotavirus and adenovirus kit tests (CerTest; Biotec, Zaragoza, Spain) were used for viral detection [22], and stool cultures were performed, when requested by the treating physician, by the direct and indirect culture methods [20].

### The sample size and power of the study

The level of confidence in this study was set at 95% with alpha error = 0.05. With a previously detected prevalence of AGE in inpatient cases at our locality of 40.4% [20], the power of this study was settled at 90% with beta error of 0.10. The estimated sample size was 370. The research team decided to increase the sample size by adding 249 (Total number 619) patients to increase the power of the study.

### Statistical analysis

Statistical Package for Social Science software (SPSS, Inc.), version 20.0, was used for conducting the statistical analyses. This software was used as well for data management and cleaning. Descriptive statistics were carried out and reported as frequencies and percentages for categorical variables, and as means and standard deviation (±) for continuous ones. After tabulating the patients’ clinical characteristics, baseline comparisons between the five studied groups were performed using Kruskall Wallis test for continuous variables. Chi-square test was used to evaluate any significant difference between the categorical variables. Associations between the infectious agents (unidentified pathogens, rotavirus, *E. histolytica,* adenovirus, and mixed enteropathogens) as dependent variables on one hand, and breast-feeding, rotavirus vaccine, intake of sanitary water, and age as independent variables on the other hand, were determined using five separate logistic regression models, a model for each infectious agent. The level of significance was set at *P* < 0.05 for all statistical analyses.

## Results

### Socio-demographic characteristics

During the period between January 2017 and December 2017, out of 3058 Lebanese patients admitted to pediatrics departments of three tertiary healthcare centers in South-Lebanon, 619 patients suffering from acute gastroenteritis (AGE) were diagnosed due to different enteropathogens. Among those, 42.7% were females and 57.3% were males. Patients were divided into six age groups between 1 and 60 months (1–3, 4–11, 12–23, 24–35, 36–47, 48–60), as previously described [13]. The mean family size of patients was 4.1, 76.4% of them had intake of sanitary water, 25.9% of mothers were defined as housewives, 68.5% of patients were breastfed, and 53.5% had taken the rotavirus vaccine.

**Fig. 1:**
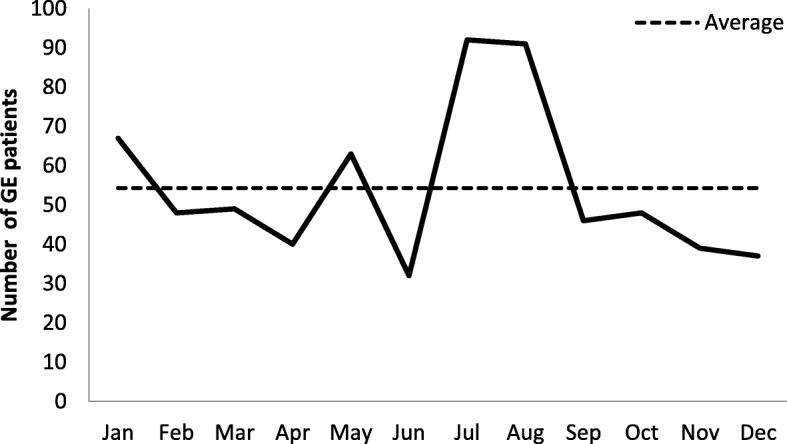
Number of acute gastroenteritis patients according to the month of admission

Regarding the monthly distribution of AGE cases, the highest number was clearly observed during the warm period especially between July and August (92 and 91 cases, respectively) of hospitalized children (Fig. 1).

### Enteropathogenic causes

Different groups of enteropathogens were detected among patients. *E. histolytica* was the lead known enteropathogen with 27.8% of cases, followed by rotavirus, adenovirus and Mixed group (two or more identified enteropathogens) with 13.6, 6.1 and 6.1%, respectively. 46.4% of cases were classified as unidentified enteropathogens and this might be due to the absence of advanced bacterial diagnosis and lack of detection of some viruses (astrovirus and norovirus) and protozoan parasites (*Giardia lamblia*). In fact, bacterial diagnosis was done just when it was requested by the treating physician (in 11.3% of cases).

### Demographic characteristics among the five studied groups

The distribution of age revealed that 55.9% (346/618) of our patients were aged between 4 and 23 months. The distribution of age groups among the different groups of enteropathogens was highly remarkable in children aged between 4 and 23 months (*P* = 0.031). Concerning the sex distribution of our patients, even in the presence of a slight difference between the percentages of the two sexes in case of unidentified group, no significant difference was observed between the five groups. Among the different demographic characteristics, patients who were breastfed, rotavirus immunized (*P* < 0.001) and patients who take sanitary water had the highest percentages of infection by unidentified agents or *E. histolytica* (Table 1).

**Table 1:** Demographic characteristics of patients among the five studied groups

Demographic Characteristics	Unidentified group I n/N (%)	*rotavirus* group II n/N (%)	*E. histolytica* group III n/N (%)	*adenovirus* group IV n/N (%)	Mixed group V n/N (%)	Total GE N	*P*-value
Age (months)							**0.031**
1–3	28/52 (53.8%)	5/52 (9.6%)	16/52 (30.8%)	2/52 (3.8%)	1/52 (1.9%)	52	
4–11	74/178 (41.6%)	26/178 (14.6%)	50/178 (28.1%)	12/178 (6.7%)	16/178 (9.0%)	178	
12–23	76/168 (45.2%)	26/168 (15.5%)	46/168 (27.4%)	7/168 (4.2%)	13/168 (7.7%)	168	
24–35	50/94 (53.2%)	11/94 (11.7%)	22/94 (23.4%)	9/94 (9.6%)	2/94 (2.1%)	94	
36–47	26/57 (45.6%)	12/57 (21.1%)	11/57 (19.3%)	7/57 (12.3%)	1/57 (1.8%)	57	
48–60	33/69 (47.8%)	4/69 (5.8%)	27/69 (39.1%)	1/69 (1.4%)	4/69 (5.8%)	69	
Total	287	84	172	38	37	618	
Gender							0.250
Female	111/266 (38.7%)	36/266 (42.9%)	85/266 (49.4%)	16/266 (42.1%)	18/266 (47.4%)	266	
Male	176/353 (61.3%)	48/353 (57.1%)	87/353 (50.6%)	22/353 (57.9%)	20/353 (52.6%)	353	
Total	287	84	172	38	38	619	
Breast feeding							0.094
No	84/175 (48.0%)	25/175 (14.3%)	36/175 (20.6%)	15/175 (8.6%)	15/175 (8.6%)	175	
Yes	169/381 (44.4%)	55/381 (14.4%)	115/381 (30.2%)	20/381 (5.2%)	22/381 (5.8%)	381	
Total	253	80	151	35	37	556	
Rotavirus vaccine							**0.000**
No	108/256 (42.2%)	53/256 (20.7%)	66/256 (25.8%)	7/256 (2.7%)	22/256 (8.6%)	256	
Yes	142/295 (48.1%)	27/295 (9.2%)	84/295 (28.5%)	27/295 (9.2%)	15/295 (5.1%)	295	
Total	250	80	150	34	37	551	
Sanitary water							0.333
No	70/146 (47.9%)	15/146 (10.3%)	41/146 (28.1%)	13/146 (8.9%)	7/146 (4.8%)	146	
Yes	217/473 (45.9%)	69/473 (14.6%)	131/473 (27.7%)	25/473 (5.3%)	31/473 (6.6%)	473	
Total	287	84	172	38	38	619	
Housewife mothers							0.843
No	187/413 (45.3%)	56/413 (13.6%)	114/413 (27.6%)	27/413 (6.5%)	29/413 (7.0%)	413	
Yes	67/144 (46.5%)	24/144 (16.7%)	37/144 (25.7%)	8/144 (5.6%)	8/144 (5.6%)	144	
Total	254	80	151	35	37	557	
Mean Family size	4.02	4.45	4.04	3.64	4.34	4.1^a^	**0.026**

^a^Mean of the five groups; significant *p*-values are made bold

**Fig. 2:**
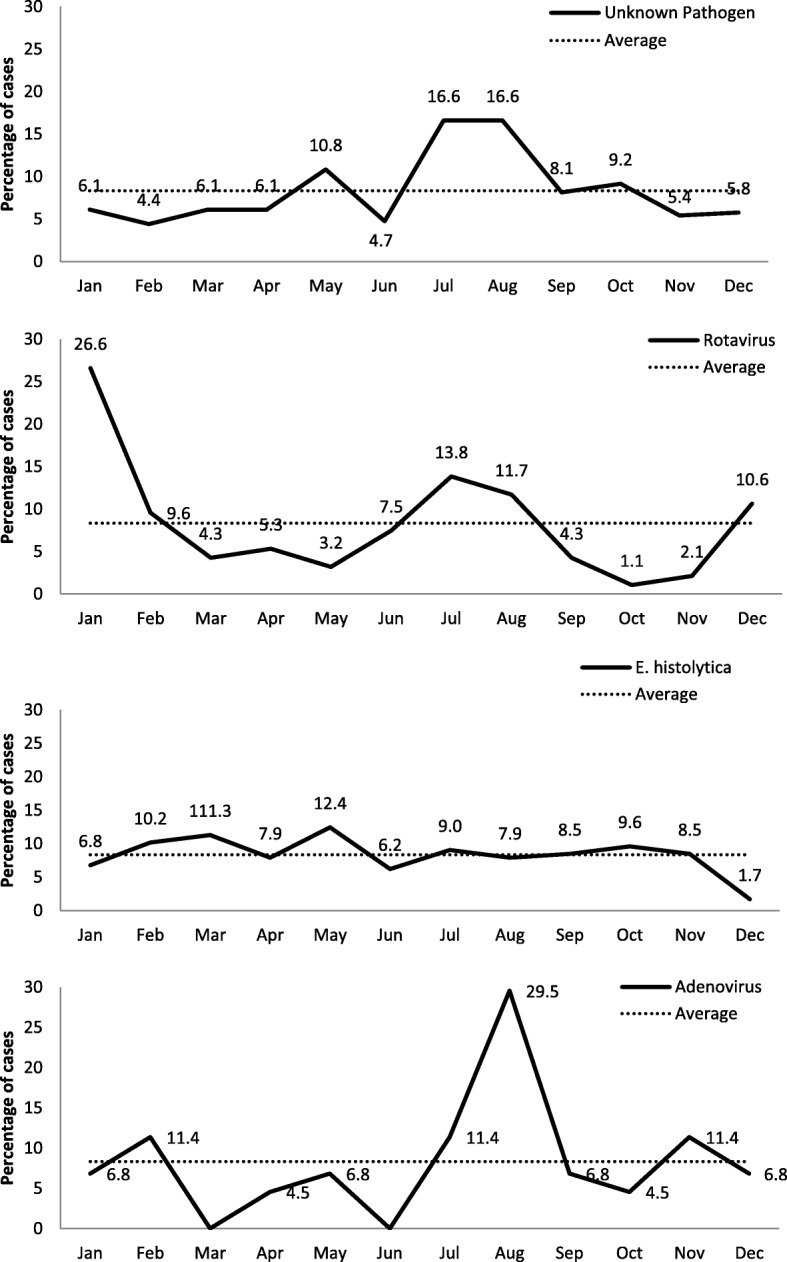
Percentage of cases of each enteropathogen according to the month of admission

Regarding the monthly distribution of each enteropathogen in this study, our results showed that rotavirus is more prevalent in January compared to its yearly average, whereas the unidentified group had an important peak in July and August, compared to its yearly average. A significant peak was also observed in August for adenovirus group. Concerning *E. histolytica*, it had small fluctuations around its yearly average (Fig. 2).

### Clinical characteristics among the five studied groups

Table 2 shows the clinical and laboratory data of patients among the five studied groups. The most common sign between the five groups was diarrhea 78.0%, followed by fever 74% and vomiting 44.7%. Fever was significantly higher (*P* = 0.001) in both unidentified and *E. histolytica* groups compared to the three other groups (74.9 and 83.1%, respectively), whereas vomiting was significantly higher in viral and mixed groups (*P* < 0.001). There was a significant difference between the means of Vesikari score (*P* = 0.005) in the five studied groups. Regarding diarrhea and duration of hospitalization, significant differences were observed among the five groups (*P* = 0.016 and *P* = 0.003, respectively) (Table 2).

**Table 2:** Clinical and laboratory data of patients

Clinical Characteristics	Unidentified group I	*rotavirus* group II	*E. histolytica* group III	*adenovirus* group IV	Mixed group V	Total	*P*-value
Frequency (%)	287 (46.4%)	84 (13.6%)	172 (27.8%)	38 (6.1%)	38 (6.1%)	619	–
Clinical Manifestations							
Fever	215 (74.9%)	52 (61.9%)	143 (83.1%)	22 (57.9%)	26 (68.4%)	458	**0.001**
Vomiting	112 (39.0%)	53 (63.1%)	66 (38.4%)	22 (57.9%)	24 (63.2%)	277	**0.000**
Diarrhea	208 (72.5%)	69 (82.1%)	144 (83.7%)	33 (86.8%)	29 (76.3%)	483	**0.016**
Vesikari score (Mean ± SD)	11.2 ± 1.8	11.4 ± 1.5	11.8 ± 1.6	11.2 ± 1.6	11.3 ± 1.8	–	**0.005**
Duration of hospitalization per days (Mean ± SD)	2.7 ± 1.2	2.9 ± 1.1	2.9 ± 1.2	3.3 ± 2.1	3.4 ± 1.3	3.04^a^	**0.003**
Laboratory Findings							
WBCs (× 10^3^ per mm^3^)	2.79 ± 3.85	2.29 ± 3.04	12.23 ± 4.98	1.92 ± 2.29	7.15 ± 6.76	–	**0.000**
RBCs (million per mm^3^)	1.47 ± 1.88	1.53 ± 2.06	7.10 ± 5.45	1.14 ± 1.01	3.19 ± 3.99	–	**0.000**
HGB (in mg/dL; mean ± SD)	11.7 ± 2.4	11.5 ± 1.2	12.6 ± 8.4	12.1 ± 1.0	11.5 ± 1.1	–	**0.008**
HCT (in %; mean ± SD)	33.5 ± 4.1	33.9 ± 3.2	34.0 ± 4.2	34.8 ± 4.7	34.1 ± 2.9	–	**0.027**
BS (in mg/dL; mean ± SD)	91.1 ± 28.3	77.1 ± 20.1	97.5 ± 19.2	86.0 ± 27.7	87.3 ± 19.1	–	**0.000**
CRP (in mg/dL; mean ± SD)	49.5 ± 65.9	28.0 ± 61.4	66.3 ± 67.4	11.7 ± 20.0	43.7 ± 85.9	–	**0.000**

Abbreviations: *WBCs* White blood cells, *RBCs* Red blood cells, *HGB* Hemoglobin, *HCT* Hematocrit, *BS* Blood sugar, *CRP* C-reactive protein

^a^Mean of the five groups; significant *p*-values are made bold

Laboratory findings presented significantly higher average of WBC, RBC, HGB, BS and CRP (> 30 mg/dL highly positive) in *E. histolytica* than the 4 other groups (*P* < 0.001, *P* < 0.001, *P* = 0.008, *P* < 0.001 and *P* < 0.001, respectively) (Table 2).

### Effect of breast feeding, rotavirus vaccination, and sanitary water on different pathogens

We analyzed the protective factors from enteropathogens associated with AGE. Results showed that in general, breastfed children might be less prone to unidentified enteropathogens, adenovirus and mixed infections. On the other hand, the rotavirus vaccine significantly protects against rotavirus (*P* < 0.001). However, the children given rotavirus vaccine had AGE attributed to *E. histolytica,* adenovirus and other enteropathogens not targeted in the study setting. Drinking sanitary water was associated with a lower frequency of unidentified enteropathogens, *E. histolytica* and adenovirus (Table 3).

**Table 3:** Results of the logistic regression analysis with infectious agents as dependent variables according to breast-feed, Rota-vaccine, and sanitary water

Independent variables	Unidentified group I		*rotavirus* group II		*E. histolytica* group III		*adenovirus* group IV		Mixed group V	
	OR	95% CI	OR	95% CI	OR	95% CI	OR	95% CI	OR	95% CI
Breast-feed*	0.866	0.606-	1.013	0.608-	1.668	1.089-	0.592	0.295-	0.655	0.331-
*p*-value	0.431	1.238	0.959	1.688	**0.019**	2.555	0.139	1.185	0.223	1.294
Rotavirus vaccine*	1.224	0.875-	0.377	0.229-	1.114	0.765-	3.505	1.500-	0.558	0.283-
*p*-value	0.238	1.712	**0.000**	0.621	0.573	1.624	**0.004**	8.191	0.092	1.100
Sanitary water*	0.930	0.643-	1.500	0.830-	0.988	0.654-	0.574	0.286-	1.400	0.603-
*p*-value	0.700	1.346	0.180	2.710	0.955	1.92	0.119	1.153	0.433	3.249

*Reference groups: Breast-feed (Yes), Rota-vaccine (Yes), and Sanitary water (Yes); significant *p*-values are made bold

### Pathogens predisposition according to age

It was found that rotavirus increased significantly in children from 12 to 23 months and children from 36 to 47 months (*P* = 0.047 and *P* = 0.014 respectively) while adenovirus increased significantly only in children from 36 to 47 months (*P* = 0.035) compared to the reference age group (48–60). However, children between 24 to 35 and children between 36 to 47 months were less prone to have *E. histolytica* in comparison to the reference age group (48–60) (*P* = 0.035 and *P* = 0.023 respectively). Results are shown in Table 4.

**Table 4:** Results of the logistic regression analysis with infectious agents as dependent variables according to different age groups

Infectious Agents	Age groups (in months)					
	1–3	4–11	12–23	24–35	36–47	48–60
Unidentified group I: OR (95% CI); *P*-value	1.343 (0.656–2.753); 0.420	0.819 (0.471–1.425); 0.480	0.931 (0.534–1.623); 0.801	1.252 (0.677–2.314); 0.474	0.966 (0.480–1.944); 0.922	1.000
*rotavirus* group II: OR (95% CI); *P*-value	1.782 (0.454–6.989); 0.407	2.865 (0.962–8.532); 0.059	3.024 (1.015–9.012); **0.047**	2.168 (0.661–7.113); 0.202	4.467 (1.355–14.726); **0.014**	1.000
*E. histolytica* group III: OR (95% CI); *P*-value	0.724 (0.339–1.547); 0.405	0.637 (0.356–1.137); 0.127	0.605 (0.336–1.087); 0.093	0.484 (0.247–0.952); **0.035**	0.390 (0.173–0.879); **0.023**	1.000
*adenovirus* group IV: OR (95% CI); *P*-value	2.800 (0.247–31.733); 0.406	5.060 (0.646–39.664); 0.123	3.006 (0.363–24.893); 0.307	7.241 (0.896–58.535); 0.063	9.800 (1.169–82.176); **0.035**	1.000
Mixed group V: OR (95% CI); *P*-value	0.328 (0.036–3.028); 0.326	1.654 (0.533–5.132); 0.383	1.387 (0.436–4.409); 0.579	0.356 (0.063–2.002); 0.241	0.299 (0.032–2.754); 0.287	1.000

Significant *p*-values are made bold

## Discussion

Most major health concerns among children under 5 years of age in Lebanon and other developing countries are respiratory and diarrheal diseases such as acute gastroenteritis (AGE) [1]. The etiologies of AGE comprise a long list of viral, parasitic and bacterial pathogens that have been identified in infected individuals. Identifying the enteropathogens that account for AGE is crucial for the application of suitable public and clinical procedures to control the disease [23]. This study was performed for a period of 1 year between January and December 2017 and covered a large area of Southern Lebanon. To the best of our knowledge, this is one of few studies conducted in Lebanon to determine the pathogens causing AGE among Lebanese children below the age of five and to check whether there exists a relation between the different etiologies of the illness and its severity and seasonal variations, using routine common laboratory methods. In our study, we included 619 Lebanese pediatric patients hospitalized with AGE out of 3058 patients admitted to the pediatrics departments during that period.

Among all the AGE hospitalized pediatric patients, we were able to identify the enteropathogen causing the illness in 53.6% of the cases only, which is less than the 67% identified by Valenzuela et al. in Chile in 2018, however they used the Film Array Gastrointestinal panel diagnostic technique [24]. Data from other Middle Eastern countries showed AGE causing enteropathogens’ prevalence rates of 28% in Bahrain [25], 63% in Palestine [26], 53.4% in Saudi Arabia [27], and 57.6% in Lebanon [20]. Out of the 332 (53.6%) detected pathogen-infected AGE cases, 88.55% (*n* = 294/332) were due to a single pathogen infection while 11.45% (*n* = 38/332) were due to mixed pathogens, which is higher than the number of co-infections obtained by an Italian study in 2018 (2.3%) [28] and much lower than the number of concurrent infections provided by Shrivastava et al. from Odisha, India in 2017 (33.8%) [29].

Of all the pathogenic agents detected in our study, *E. histolytica* was the lead AGE-causing enteropathogen with 27.8% of the cases (*n* = 172 of 619), which is very close to the results obtained in our previous study (26.3%) [20] and similar to the prevalence reported previously from Lebanon in 2013 (22.3%) [14]. Our findings also supported previous regional studies as the prevalence of *E. histolytica* among individuals (regardless of their age) was found to be 20.0% in Saudi Arabia [16] and 19.9% in Libya [30]. Moreover, the age distribution of *E. histolytica* infection was as follow: 65.1% (112/172) below 2 years of age; these results are uncommon in this age group since *E. histolytica* is usually transmitted via the fecal oral route with contaminated food and water, so young children are less prone to develop such infection regularly [31]. This may indicate that the drinking water used for milk preparation and the tap water used for daily home and body hygiene might be contaminated and orally ingested by babies during bathing or face washing. In fact, low socio-economic status is the most important demographic factor linked to the high occurrence of *E. histolytica* among children and this is probably due to low level of public and individual hygiene [32].

Concerning clinical manifestations, 83.1% of the patients with amoebiasis had high fever and severe diarrhea, and 38.4% complained of vomiting which are significantly higher compared to patients from the rotavirus group, adenovirus group and the mixed group (*p* = 0.001 for fever, *p* = 0.016 for diarrhea, and *p* < 0.001 for vomiting). Naous et al. in 2013 showed that 94.2% of patients infected with *E. histolytica* had fever [14]. Furthermore, we have found that this group has the highest Vesikari score (11.8 ± 1.6) compared to the other groups (*p* = 0.005). In addition, this group showed the highest CRP level (66.7 mg/dL) when compared to other groups (*p* < 0.001) and this is a common finding since *E. histolytica* infection is linked to high CRP levels due to the pathogen’s invasive nature [33]. This indicates that among our 4 groups of identified pathogens, *E. histolytica* is responsible for the most severe AGE. Regarding the monthly distribution of *E. histolytica*, it was shown to have small fluctuations around its yearly average with two short peaks during March (11.3) and May (12.4) indicating that the chances of *E. histolytica* infection are similar throughout the year.

The second major enteropathogen identified in our study was rotavirus, responsible for 13.6% (*n* = 84/619) of the cases diagnosed with AGE which is not far from the 19.6% previously reported in 2017 in Saudi Arabia [27] and lower than the previous reported results (26%) in 2017 in India [29]. Rotavirus is responsible for the hospitalization of more than 2 million individuals and for the death of over half a million patients form AGE in infants and young children worldwide, especially in developing countries in Africa and Asia [34].

Concerning age distribution, 13.6% (n = 84/619) of our reported AGE cases below 5 years of age were due to rotavirus which is in line with several studies from the Eastern Mediterranean region [35, 36]. Compared to its yearly average, rotavirus is more prevalent in January with a total of 27 cases admitted to the hospital during that month. In fact, several studies have shown higher rotavirus AGE incidence during the period between January and June [37]. In general, rotavirus is known to cause diarrheal illness throughout the year but predominantly during winter months in countries with temperate climates such as Lebanon. Like other diarrheal viruses, rotavirus spreads mainly through the contact with contaminated surfaces, ingestion of contaminated food or water and contact with infected persons [38].

Adenoviruses are abundant agents known to cause digestive infection in children under the age of 5, usually due to poor personal hygiene or the ingestion of contaminated food or water [39]. In our study, we have identified the presence of adenovirus as a single agent in 6.1% of the cases, a percentage that is equal to that of the mixed group and similar to the adenovirus prevalence reported in a previous study from Korea in 2017 [40]. Our results demonstrated a significant high prevalence of adenoviral infections in August represented with a major peak of 29 cases reported during that month; this peak during the summer season may be due to certain viral characteristics such as environmental stability, heat resistance, easy transmission by the fecal-oral route and more likely due to the increased intake of water during this period which could be contaminated.

Regarding the various clinical manifestations, only 57.9 and 61.9% of the cases in both the adenovirus and the rotavirus groups were diagnosed with high grade fever, respectively, and a very low CRP value (28.0 mg/dL for rotavirus and 11.7 mg/dL for adenovirus) which are less than the values observed in either the *E. histolytica* group or the unidentified group. Although similar to groups I (Unidentified group) & III (*E. histolytica* group), 82.1% of the cases infected with rotavirus and 86.8% of the cases infected with adenovirus were diagnosed with diarrhea which is considered a hallmark of AGE. Considering the Vesikari score, we found that both groups II (*rotavirus* group) and IV (*adenovirus* group) had scores below that of group III (*E. histolytica* group) indicating that rotavirus and adenoviral infection cause AGE with lower severity when compared to other enteropathogens.

In our study, we have recognized 46.4% of cases with unidentified causes or pathogens, a notable percentage that we must depend on to initiate further needed tests to improve our pathogen identification ability. Clinical manifestations of patients within this group have shown a strong prevalence of high-grade fever (74.9%) and diarrhea (72.5%) with an approximately high CRP value (49.5 mg/dL), all of which highly suggest the possibility of invasive infections as major AGE-causing enteropathogens within the group [33]. Despite the potentially low Vesikari score (11.2) tabulated for this group compared to the other groups, Vesikari score of 11 or more still indicates severe AGE. In fact, unidentified pathogens can include different viral strains, bacteria, and parasites, each of which may cause AGE with a certain severity depending on the causing agent. As a result, to improve our pathogen detection ability, we need to widen our test panel by increasing the number of cultures performed for hospitalized children with suspected invasive AGE in order to foster more pathogenic bacteria such as *Salmonella*, *Campylobacter*, *Shigella* or to cultivate more pathogenic viruses such as norovirus, astrovirus or to detect other pathogenic parasites such as *Giardia lamblia* and *Cryptosporidium* species, all of which can be potential causes of AGE within the unidentified pathogen group. A recent study by Ibrahim *el al.* showed, by using of microbiological and molecular diagnosis techniques, a prevalence of 21.5% of *Campylobacter* species in stool of Lebanese patients with AGE. This type of diagnosis must be implicated as a routine diagnosis test in future studies [41].

The average length of hospital stays (LOS) is 3.04 days and it was approximately similar in the different groups. Furthermore, our study showed that breastfed children might be less prone to unidentified enteropathogens, adenovirus and mixed infections. However, breastfeeding did not affect their susceptibility for rotavirus infection (OR = 1.013, 95% CI [0.608–1.688], *p* = 0.959) while it was found to be associated with high rates of *E. histolytica* infection (OR = 1.668, 95% CI [1.089–2.555], *p* = 0.019). Despite this, it is known that colostrum and mature human milk can significantly kill *E. histolytica* by bile salt-stimulated lipase in human milk that kills both parasites; *Giardia lamblia* and *E. histolytica* [42]. Such result may be attributed to insufficient maternal hygiene or associated improper feeding practices. Finally, drinking sanitary water had no protective effect on any given group, and this may be due to use of contaminated tap water for daily cooking and personal hygiene.

### Study limitations

We believe that our study has a number of limitations. First, the diagnostic tests were limited to the routine ones that are dependent on their availability in the participating tertiary healthcare hospitals of our study. Most of the time, the bacterial coproculture was not requested routinely on admission (it was only done if requested by the treating physician (in 11.3% of cases)). Second, the low sensitivity of microscopy in differentiating *E. histolytica* from other morphologically-similar amoebae like *E. dispar* and *E. moshkovskii* is also considered as a limitation. In fact, although WHO states that *E. histolytica* stool antigen detection test is more specific for the pathogenic amoeba *E. histolytica* than the classic stool ova and parasite examination, the latter was the one still utilized in almost all healthcare centers in South Lebanon, including the three tertiary healthcare centers in our study, which reflects the importance of improving health services in this unfortunate area of the world (South Lebanon). Third, we believe that we have an important group of unidentified pathogens that should solicit us to expand our diagnostic arsenal. Lastly, some data were missing from the medical records of the patients, such as details about breastfeeding (exclusive or not, and duration of exclusive breastfeeding).

## Conclusion

In conclusion, increasing the size of the AGE diagnostic panel may allow us to detect specific pathogens causing both invasive and non-invasive entero-colitis in hospitalized children. Consequently, we will be able to prescribe the specific treatment for each case alone. Providing a personalized treatment depending on the exact cause of infection is considered a more efficient compared to the prescription of the broad-spectrum antibiotics.

## Abbreviations

AGE: Acute gastroenteritis
BS: Blood sugar
CRP: C-reactive protein
EMB: Eosin methylene blue
HCT: Hematocrit
HGB: Hemoglobin
IRB: Institutional Review Board
LOS: The average length of hospital stays
LU: Lebanese University
SPSS, Inc.: Statistical Package for Social Science software
SS: *Salmonella-Shigella*
WHO: World Health Organization
